# Advanced Glycation End Products in the Skin: Molecular Mechanisms, Methods of Measurement, and Inhibitory Pathways

**DOI:** 10.3389/fmed.2022.837222

**Published:** 2022-05-11

**Authors:** Chun-yu Chen, Jia-Qi Zhang, Li Li, Miao-miao Guo, Yi-fan He, Yin-mao Dong, Hong Meng, Fan Yi

**Affiliations:** ^1^Beijing Key Laboratory of Plant Resources Research and Development, Beijing Technology and Business University, Beijing, China; ^2^Key Laboratory of Cosmetic, China National Light Industry, Beijing Technology and Business University, Beijing, China; ^3^Institute of Cosmetic Regulatory Science, Beijing Technology and Business University, Beijng, China

**Keywords:** advanced glycation end products, skin barrier, keratinocytes, fibroblasts, protein cross-linking, matrix metalloproteinase, measurement methods

## Abstract

Advanced glycation end products (AGEs) are a series of stable compounds produced under non-enzymatic conditions by the amino groups of biomacromolecules and the free carbonyl groups of glucose or other reducing sugars commonly produced by thermally processed foods. AGEs can cause various diseases, such as diabetes, atherosclerosis, neurodegeneration, and chronic kidney disease, by triggering the receptors of AGE (RAGEs) in the human body. There is evidence that AGEs can also affect the different structures and physiological functions of the skin. However, the mechanism is complicated and cumbersome and causes various harms to the skin. This article aims to identify and summarise the formation and characteristics of AGEs, focussing on the molecular mechanisms by which AGEs affect the composition and structure of normal skin substances at different skin layers and induce skin issues. We also discuss prevention and inhibition pathways, provide a systematic and comprehensive method for measuring the content of AGEs in human skin, and summarise and analyse their advantages and disadvantages. This work can help researchers acquire a deeper understanding of the relationship between AGEs and the skin and provides a basis for the development of effective ingredients that inhibit glycation.

## Introduction

Advanced glycation end products (AGEs) are brown substances formed in the late stage of glycation reaction between glucose or other reducing sugars and free amino groups in proteins, nucleic acids or lipids. They were first proposed by the French chemist Maillard in 1912 (1). The accumulation of AGEs is associated with the development or exacerbation of many degenerative processes or diseases (2), including diabetes (3), cardiovascular disease (4), cataracts (5), Alzheimer’s disease (6), etc., and exacerbations involve Pathology of oxidative stress mechanisms and accelerated ageing processes (7). In 1981, Monnier and Cerami (8) discovered that glycation is related to the living system. Skin interstitial tissue and collagen accelerate ageing under the influence of non-enzymatic browning, which has attracted widespread attention. Multiple comprehensive studies have found that AGEs strongly affect the dynamic balance of the skin and are a pathogenic factor for skin complications of chronic metabolic diseases (9), such as diabetic skin ulcers, infections, and non-healing wounds (10), and are the reason for the frequent occurrence of skin problems in the modern population. Studies have shown that (11–13) with the increase in the AGE content in the skin, volunteers developed skin problems such as yellowing, browning, poor elasticity, and deeper wrinkles. Researchers gradually understood that the effect of AGEs on the skin should not be underestimated. This article summarises the molecular mechanisms by which AGEs induce skin problems from the perspective of skin composition and structure, provides a systematic and comprehensive method for measuring the human skin AGE content, summarises and analyses the advantages and disadvantages of these methods, and finally discusses the prevention and inhibition of AGEs. It provides theoretical basis and new insights for researchers to develop AGE inhibitors that will relieve skin lesions of patients and improve the skin condition of the population.

## Brief Biochemistry of Advanced Glycation End Products

### Formation of Advanced Glycation End Products

**FIGURE 1:**
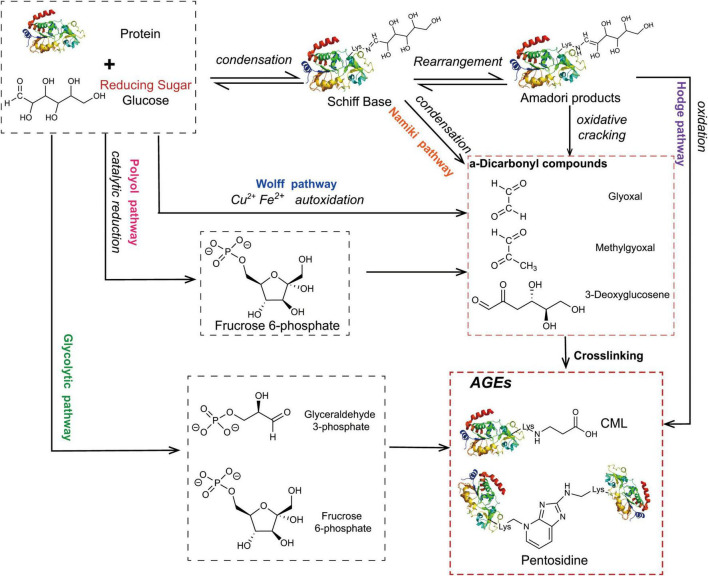
The main pathway for the formation of AGEs. The condensation of the carbonyl group of the reducing sugar with the amino acid carbonyl amine forms a Schiff base, which is rearranged by an Amadori reaction to form a stable Amadori product. Some Amadori products are converted to AGEs by the Hodge pathway, and others are oxidized and cleaved to active dicarbonyl compounds. Active dicarbonyl compounds are further cross-linked with proteins to generate AGEs. These carbonyl intermediates are also generated by the Wolff pathway, Namiki pathway, and Polyol pathway.

The Maillard reaction is the main pathway for the formation of AGEs (Figure 1), and it is divided into three stages. The first stage is the condensation of the carbonyl group of the reducing sugar with the amino acid carbonyl amine to form a Schiff base, which is rearranged by an Amadori reaction to form a stable Amadori product (14). Amadori products undergo rearrangement and degradation and are converted into highly reactive dicarbonyl compounds such as methylglyoxal (MGO), glyoxal (GO), and 3-deoxyglucosone (3-DG) (15). These carbonyl intermediates directly interact with free amino groups in proteins, resulting in a high degree of physiological damage (16). In addition to the aforementioned pathways, these carbonyl intermediates are also generated by the Wolff pathway, namely, the metal-catalysed auto-oxidation and dehydration of glucose (17). Moreover, unstable Schiff bases generate GO, MGO, and 3-DG through reverse aldol condensation and oxidative decomposition, also known as the Namiki pathway (18, 19). In organisms, dicarbonyl compounds are produced through physiological metabolic pathways, such as the formation of tricarbon phosphate and fructose-3-phosphate from glucose via the polyol pathway, which is degraded to form MGO and 3-DG (20). Alternatively, MGO, GO, and other substances are produced through lipid peroxidation. Intermediates (such as fructose 6-phosphate, glucose 6-phosphate, etc.) produced by the glycolytic pathway are also involved in the formation of dicarbonyl compounds (21). The final formation of AGEs is mainly mediated by two pathways. The first pathway is that Amadori products directly generate AGEs through the Hodge pathway, namely, through oxidative degradation or oxidative rearrangement (22), mainly including [*N*-(carboxymethyl)lysine] (CML), pentosidine, and glucosepane. The second pathway is that dicarbonyl compounds react directly with lysine residues and arginine residues on proteins to form AGEs. Notably, MGO production also increases AGE-derived cross-linking under conditions of high oxidative stress (23).

### Structure and Properties of Advanced Glycation End Products

**FIGURE 2:**
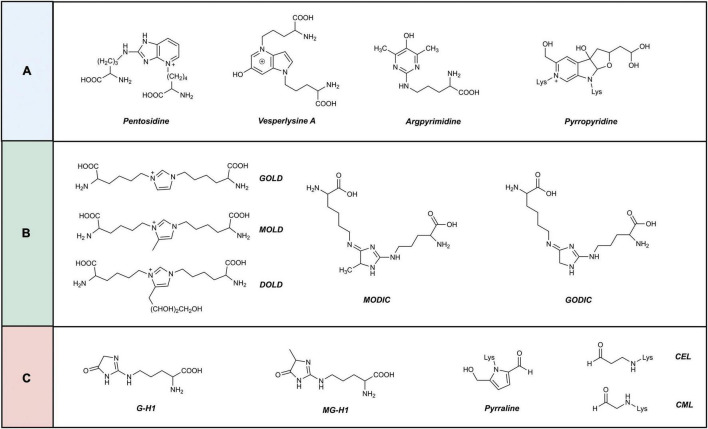
Classification of advanced glycation end products. AGEs are classified according to their crosslinking structure and fluorescence characteristics into three categories. **(A)** Fluorescent, cross-linked. **(B)** Non-fluorescent, cross-linked. **(C)** Non-fluorescent, non-cross-linked.

At present, approximately 20 AGEs have been detected in human skin (24). AGEs are classified according to their cross-linking structure and fluorescence characteristics (25) into three categories (Figure 2): (1) fluorescent, cross-linked; (2) non-fluorescent, cross-linked; and (3) non-fluorescent, non-cross-linked types. Four of them, pyrraline, pentosidine, CML, and carboxyethyl-lysine (CEL), have been extensively studied as important AGEs (26, 27).

### Sources of Advanced Glycation End Products in the Skin

The sources of AGEs in the skin are mainly divided into endogenous production and exogenous intake. Endogenous AGEs are produced and accumulate in the skin during normal physiological metabolism with ageing, or in diseases associated with inflammatory responses or chronic metabolic disorders. Exogenous AGEs are derived from the diet, cigarette smoke, ultraviolet light, and air pollution.

#### Endogenous Advanced Glycation End Products

Conventional endogenous AGEs are spontaneously formed and accumulate in the body under physiological metabolic conditions during normal ageing, and the reaction process is slow due to the absence of enzymes (3). The levels of AGEs (CML, CEL, and pentosides) in skin collagen increase linearly with age (28–30). Verzijl et al. (30) showed that the long turnover time of collagen in skin is the main reason for the accumulation of AGEs.

Inflammatory reactions or chronic metabolic disorders such as diabetes mellitus (DM) and chronic renal failure are important sources of endogenous AGEs. The long-term high blood sugar state in patients with DM is a key factor promoting the formation of AGEs (31), and the production and accumulation of AGEs is one of the main mechanisms of diabetes complications (32). In patients with renal failure, reduced elimination of water-soluble, low-molecular weight AGEs and high levels of oxidative stress also contribute to the accumulation of AGEs (33). Some scholars proposed the theory of “common soil” (34–36). AGEs accumulate in large quantities in organisms and “guide” obesity, diabetes and diabetic complications by inducing insulin resistance syndrome (IR), producing metabolic disorders and pathogenic environmental factors. Endogenous AGEs appear faster and accumulate more extensively, further forming a feed forward-driven pathological cycle that mediates a series of metabolic dysfunctions.

The fluorescence level of AGEs in the skin has been shown to determine the survival rate of patients on dialysis (37, 38), is an important marker of cardiovascular mortality in patients with chronic kidney disease (CKD) (38, 39), and has been used to measure the risk of DM complications (40). The content of AGEs in the skin of haemodialysis patients is significantly higher than that of healthy individuals (33, 41). Patients with severe psoriasis (42), diabetes and insulin resistance (IR) syndrome (43, 44), and systemic lupus erythematosus (45, 46) exhibit exacerbated production of endogenous AGEs, and the levels of AGEs in their skin were higher than those in the control group due to their persistent chronic inflammation, hyperglycaemia, or oxidative stress. In clinically, due to the differences in AGEs expression among these diseases that the skin fluorescence AGEs can utilised to predict the occurrence of diseases such as diabetes, renal or cardiovascular diseases (47).

Oxidative stress is an important factor contributing to the formation of endogenous AGEs. When organisms are under oxidative stress for a long time, the body’s original defence mechanisms are exhausted and ROS are over-produced and accumulate, resulting in increased levels of reactive aldehydes and their derivatives and eventually leading to the massive production of endogenous AGEs and ALEs (advanced lipoxidative end products) (48). On the other hand, AGEs trigger various physiological and pathological responses by activating the receptor of Advanced glycation end products (RAGE) (49), and activate NADPH oxidase to increase ROS levels. ROS are involved in the process of monosaccharide autoxidation and play a role in the development and stabilisation of cross-links of early glycation products (50).

#### Exogenous Advanced Glycation End Products

The intake of exogenous AGEs mainly is mainly derived from food and is called food-derived advanced glycation end products (dAGEs). The Maillard reaction of food during high-temperature cooking (grilling, frying, baking, etc.) produces a large amount of dAGEs, which are closely related to the colour, flavour, and taste of food (51). A Rotterdam study indicated that the intake of dAGEs was positively correlated with the content of AGEs in the skin (52). With the development of society and changes in the dietary habits of the population, the consumption of this highly processed food has increased exponentially, and AGEs may be the most important factor in the link between modern diet and health (26, 53).

Cigarette smoke is also an important source of exogenous AGEs (54). Dickerson and Janda (55) showed that the tobacco metabolite nornicotine is involved in the synthesis of Amadori products, causing abnormal protein glycation, and the skin AGEs fluorescence value (SAF) of smokers is significantly higher than that of non-smokers (56). The skin AGE levels of breastfed infants in the smoking group were higher than those in the non-smoking group (57).

Ultraviolet rays and air pollution also increase the content of skin AGEs (27, 36). The AGE content increased in fibroblasts treated with fly ash simulated granular air pollution (58). The dermal CML content of sun-exposed skin is more than 10% higher than that of sun-protected skin, and UV light further promotes the accumulation of CML and pentosides in the skin by inducing oxidative stress (59, 60).

### Metabolic Pathways of Advanced Glycation End Products

Organisms activate multiple glycation defence systems to prevent AGE-mediated cytotoxicity, and protect human tissues from glycation-induced damage by promoting the metabolism of AGEs and their precursors to repair and eliminate glycation products. These pathways include the glyoxalase system, fructose amine 3-kinase (FN3K) repair enzyme, ubiquitin-proteasome system (UPS) and autophagy system (61, 62). The glyoxalase system, FN3K, reduces the formation of precursor compounds of AGEs. Glyoxalase I catalyses the transformation of dicarbonyl compounds such as MGO, GO and glutathione to D-lactyl glutathione, which is converted into the non-toxic compound D-lactate through the action of glyoxalase II and excreted (63–65). FN3K is an enzyme that repairs Amadori products. It phosphorylates fructose-conjugated lysine residues to destabilise them from proteins, thereby separating the binding of sugars to proteins to effectively deglycosylate proteins (66). The UPS and the autophagy system are pathways for the elimination of AGEs in organisms (67, 68). The UPS plays an important role in the protein quality control mechanism and is responsible for maintaining the normal functioning of cells by removing damaged proteins. The two systems independently or cooperatively remove AGEs (69), but the specific mechanism of AGE removal remains unclear (61).

## Various Advanced Glycation End Products Damage Different Levels of the Skin Structure

**FIGURE 3:**
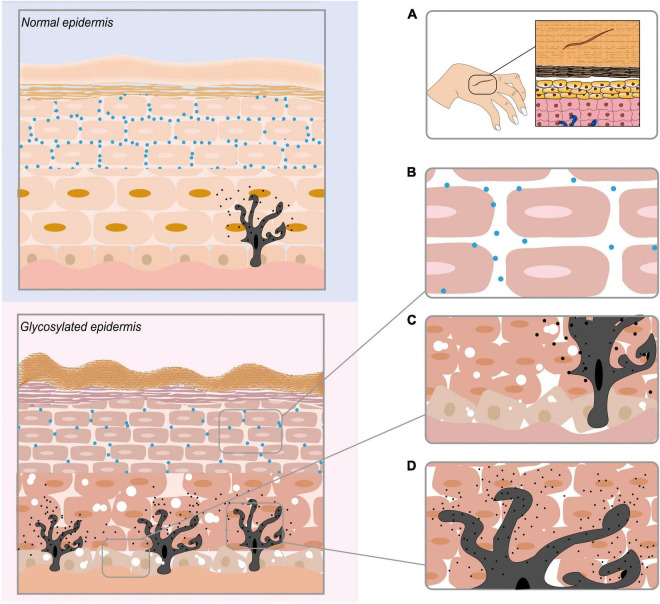
The effect of AGEs on the epidermis of the skin. **(A)** AGEs obstruct skin wound healing. **(B)** AGEs reduce the contents of ceramide (CER) and cholesterol (CHOL) in the epidermis, eventually leading to a reduction in skin lipid content. **(C)** AGEs destroy the keratinocyte cell structure in the epidermis. **(D)** AGEs promote the production of melanin in melanocytes.

**FIGURE 4:**
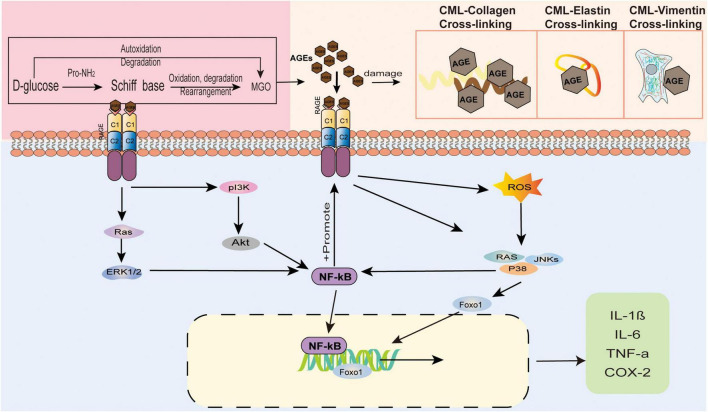
The effect of AGEs on the dermis of the skin. AGEs and collagen are cross-linked, causing the protein to brown and the fibers to deform. Elastin fiber becomes thinner, less rigid, and loses its biological properties. Glycosylated vimentin leads to the loss of fibroblasts’ contraction ability and the inability to maintain the basic cell shape. AGEs bind to RAGE receptors to regulate gene expression and mediate a series of signal pathways.

Several studies have indicated that AGEs accumulate after they are produced in the human body, which leads to the destruction of skin tissues by regulating gene expression, destroying protein structures, binding to RAGEs, mediating a series of signalling pathways, and affecting the apoptosis and differentiation of skin-related cells. AGEs affect all levels of the skin, causing inflammation, ageing, yellowing and other issues. We demonstrate the consequences of AGEs on the epidermis of the skin in Figure 3, and those of the dermis of the skin are shown in Figure 4. Table 1 summarises the effects of AGEs on each layer of the skin and the corresponding molecular mechanisms.

**TABLE 1:** The effects of advanced glycation end products (AGEs) on various layers of the skin and their molecular mechanisms.

Substance	Mechanism	Symptoms	References
Sebaceous membrane	(i) Decreased expression of ceramide synthase CERS3 reduces the content of ceramide (CER) and cholesterol (CHOL) in the epidermis (ii) Reduced epidermal lipid synthesis (iii) The function of structural proteins (filaggrin, transglutaminase-1) is affected	(i) The skin barrier is destroyed (ii) Decrease lamina, delaying the self-repair of the skin barrier (iii) The function of related proteins to establish the skin barrier is destroyed	(70–72)
Keratinocytes	(i) The stratification of keratinocytes in the epidermis is disordered and the cytoplasm is vacuolated (ii) With the reduction of epidermal lipid synthesis, the integrity of the stratum corneum decreases	(i) Loose skin structure (ii) Thinning of the epidermis	(71, 73–75)
Melanocytes	(i) AGEs bind to RAGEs, activate ERK and CREB signalling pathways, and increase MITF expression and tyrosinase activity	(i) The production of melanin in melanocytes is promoted and the skin is prone to photoaging	(76)
Epidermal ECM	(i) The RAGE-MAPK-ERK1/2 or p38 pathway upregulates the expression of MMP-9 in cells	(i) The overexpression of MMP-9 affects skin wound healing	(77)
Fibroblasts	(i) In the cell: expression of the CatD enzyme is reduced, ROS activation, expression of p38/JNK is induced, and the FOXO1 transcription factor is activated (ii) Cell membrane: The fluidity of the cell membrane and liposome membrane increases; mast cells release histamine and the production of hyaluronidase increases, and sodium hyaluronate HA reduces AGE-RAGE-MAPKs (p38, PI3K/Akt, ERK, JIKs), increases the production of ROS, activates the expression of the NF-kB transcription factor HMGB1, and after binding to RAGEs, induces the expression of the TRPV1 protein	(i) AGEs accelerate their deposition and accumulation in the skin, which further accelerates the senescence caused by photoaging; fibroblast apoptosis (ii) Inflammation (iii) Skin sensitivity to pain	(22, 78–84)
Dermis ECM	(i) The activities of the matrix metalloproteinases MMP-1, MMP-2, and MMP-9 all increase, which changes the expression of ECM-related genes in fibroblasts (ii) Cross-linking with collagen, vimentin, and elastin, and long-term protein accumulation	(i) Changes the balance between the synthesis and degradation of the extracellular matrix, which ultimately leads to impaired skin homeostasis (ii) Destruction of the protein structure and fibre deformation, making them unable to maintain their biomechanical properties and functions (iii) The browning of collagen causes skin yellowing, the loss of fibroblast contraction ability, and ultimately accelerates the ageing process (iv) Skin sensitivity to pain	(72, 74, 85–90)

### The Effect of Advanced Glycation End Products on the Epidermis

#### Advanced Glycation End Products Cause Impaired Skin Barrier Function

As a firm structure on the surface of the skin, the skin barrier has the ability to limit water loss and maintain an important shielding function and consists of the stratum corneum and sebaceous membrane. An *in vitro* study by Yokota et al. (70) showed that AGEs reduce the contents of ceramide (CER) and cholesterol (CHOL) in the epidermis by reducing the expression of ceramide synthase (CERS3). This is similar to the results of Park et al. (71)’s research based on rats. Under the stimulation of glycation, the synthesis of epidermal CHOL in rat’s decreases, which leads to a decrease in the lamina, thereby delaying the self-repair of the barrier. Studies by Lee et al. (72) have shown that glycation causes the function of epidermal structural proteins (filaggrin, transglutaminase-1) to be affected, causing damage to the skin barrier. From *in vitro* and *in vivo* experiments, there is sufficient evidence at the molecular level to show that under the influence of glycation, the skin barrier is damaged and cannot maintain its protective functions.

#### Advanced Glycation End Products Destroy the Keratinocyte Cell Structure in the Epidermis

Keratinocytes, as the main constituent cells of the epidermis, play an important role in skin health. Studies have shown that keratinocytes under the influence of glycation are disordered in the epidermal layer, which leads to cytoplasmic vacuolation (73). Experiments by Park et al. (71) showed that the integrity of the stratum corneum decreases when lipid synthesis in the epidermis is reduced. A 3D skin model after glycation showed that α1 integrin and β6 integrin in the epidermal cell basal layer were overexpressed under the influence of AGEs (74, 75). As a result, the stratification of keratinocyte cells was disordered, the cytoplasm was vacuolated, and the stratum corneum became thin.

#### Advanced Glycation End Products Promote the Production of Melanin in Melanocytes

Research by Lee et al. (76) showed that AGEs secreted by keratinocytes under ultraviolet irradiation combined with RAGEs and through the ERK and CREB signalling pathways, increased MITF expression and tyrosinase activity and ultimately promoted the production of melanin in melanocytes. The mechanism of AGEs and RAGEs may contribute to preventing photoageing.

#### Advanced Glycation End Products Obstruct Skin Wound Healing

Diabetes patients produce more glycation end products than non-diabetic individuals and often suffer from foot ulcer complications. Zhu et al. (77) studied the effect of AGEs on the production of MMP-9 in HaCaT cells, and the results showed that AGEs upregulate the expression of MMP-9 in cells through the RAGE-MAPK-ERK1/2 or p38 pathway. Further, the overexpression of MMP-9 affects skin wound healing. Pageon et al. (74) using a full-thickness restructuring skin model, also proved that the 9activity of MMP-9 was increased under AGE stimulation.

### The Influence of Advanced Glycation End Products on the Dermis

#### Advanced Glycation End Products Promote Fibroblast Apoptosis

In photoageing fibroblasts, AGEs accelerate deposition and accumulation in the skin by reducing the expression of the CatD enzyme, which reduces the ability to degrade AGEs, thereby further accelerating photoaging (78). A study showed that CML, as the most abundant AGE in the human body, activates ROS, induces the expression of p38/JNK, activates the FOXO1 transcription factor, and ultimately leads to fibroblast apoptosis (79).

The interaction between AGEs and fibroblast membranes causes changes in cell function. AGEs increase the fluidity of cell membranes and liposome membranes and change their hydrophobicity. Sodium hyaluronate (HA) decreases with increasing AGEs (80). A study in diabetic model mice pointed out that mast cells in type 2 diabetic mice release histamine and increase the production of hyaluronidase, which leads to a decrease in the synthesis of HA (81). This may explain the fact that diabetic patients are prone to suffer from dry skin under long-term high-sugar conditions.

RAGEs have the highest expression level in skin fibroblasts. In the human body, both exogenous and endogenous AGEs trigger various physiological and pathological responses by activating RAGEs. After AGEs directly cross-link or indirectly bind to cell surface receptors, they activate MAPK signalling molecules, including p38, phosphatidylinositol 3-kinase (PI3K)/Akt, MAPK/ERK, and JIKs, and the combination of AGEs and RAGEs also increases ROS production. ROS production is the most important induction mechanism of the inflammatory response. ROS activation increases the levels of the nuclear factor kB (NF-kB) transcription factor (82). RAGEs activate NF-κB through a signalling pathway (83), thereby stimulating ROS upregulation and inducing the expression of RAGEs, creating a positive feedback loop. The results of these events include upregulation of inflammation, induction of oxidative damage, interference of cell movement, and changes in cell metabolism (22, 49). In a long-term high glucose environment, HMGB1 acts as a RAGE agonist after binding, induces the expression of the TRPV1 protein, sensitises neurons, and easily induces pain (84).

#### Advanced Glycation End Products Destroy Fibre Contracture in the Dermis

Advanced Glycation End Products easily accumulate in the extracellular matrix of the dermis, changing the balance between the synthesis and degradation of the extracellular matrix and ultimately leading to impaired skin homeostasis. The recombinant skin model established by Pageon showed increased activity of matrix metalloproteinases (MMP-1, MMP-2, and MMP-9) after glycation stimulation (74). The skin model of Lee et al. (72) also confirmed that under glycation, matrix degrading enzymes (MMP-1) and extracellular matrix (ECM) synthesis (collagen, elastin, etc.) are reduced, leading to degradation of the ECM. The results of Lohwasser et al. (85) also confirmed that AGEs change the expression of ECM-related genes in fibroblasts. Under AGE stimulation, HFF produces excessive MMP-2, and the basement membrane was destroyed.

The extracellular matrix is rich in longevity proteins, collagen, vimentin, elastin, etc. After AGEs are produced, they easily bind and cross-link proteins. Under long-term accumulation, the protein structure is destroyed and the fibres are deformed, making them unable to maintain biomechanical properties and functions.

Collagen is one of the structures most easily attacked by AGEs. Due to the irreversibility of non-enzymatic cross-linking and the low turnover of collagen, AGEs gradually accumulate on collagen over time, which causes the collagen to brown and the skin to turn yellow. After collagen and AGEs are cross-linked, the fibre deformation reaches more than 80% of all tissue deformation, resulting in a loss of obvious stress relaxation behaviour (86). The full-thickness restructuring skin model of Lee et al. (88) showed that the yellowness of the skin increases with increasing AGEs (CML). Laughlin et al. (87) collected female skin samples to determine the content of AGEs, and the number of AGEs was significantly higher in people with dull and yellow skin.

Elastin cannot avoid cross-linking with AGEs. Lee et al. (88) developed a face glycation imaging system to study the correlation between facial skin elasticity and AGEs in healthy women, and the results showed that facial elasticity was negatively correlated with the cheek skin glycation index. The reasons may be thinning of the elastin fibres saccharified under confocal microscopy, a decrease in hardness, and the loss of elastin’s biological properties (89).

Kueper et al. (90) first determined that vimentin is the main target of CML in fibroblasts, and studies have shown that the accumulation of CML-vimentin can be found in the living skin fibroblasts of elderly donors. The modification of vimentin by CML leads to the loss of fibroblast contraction and ultimately accelerates the ageing process.

## Skin Advanced Glycation End Products Measurement Methods

When researching glycation reactions, experimenters usually use high-performance liquid chromatography (HPLC), gas chromatography (GC), mass spectrometry (MS), and enzyme-linked immunosorbent assays (ELISAs) to determine the content of AGEs. These methods mostly target biological samples such as serum, tissue, and urine. For more than 10 years, there have been several methods for measuring the content of AGEs in human skin, and these methods are non-invasive, real-time, and convenient. We describe and analyse the methods for measuring the content of skin AGEs and summarise the advantages and disadvantages of these methods in Table 2.

**TABLE 2:** Different methods for measuring the content of advanced glycation end products (AGEs) in human skin and their advantages and disadvantages.

Method	Material	Advantages	Disadvantages	References
Autofluorescence reader (AFR)	Diabetic human skin	1. Non-invasive, simple and fast 2. Significantly correlated with the level of AGEs measured by HPLC (accuracy)	1. Non-fluorescent AGEs cannot be detected 2. Cannot rule out interference from other fluorophores 3. Skin tone interferes with the final result	(91)
Multiphoton autofluorescence (MPAF) Second harmonic generation (SHG)	Human skin without a history of diabetes	1. Real-time monitoring of the spatial and temporal effects of glycosylation on skin tissue 2. Imaging of the epidermis, collagen, and elastic fibres 3. Imaging and quantification of deep skin glycosylation	1. The instrument is expensive 2. The experiment is complex and requires highly technical personnel	(92)
Confocal Raman spectroscopy (CRS)	Human skin under high ultraviolet radiation	1. Real-time and non-invasive 2. Full-thickness skin tissue (epidermal layer, dermis layer) can be measured 3. The affected molecular mechanism can be explored through spectral changes	1. The instrument is expensive and requires strict operation by the staff 2. Low recognition rate of AGEs in the dermis	(93)
Confocal Raman microspectroscopy(CRM)	Collagen scaffold for diabetic mice	1. For the first time, can be used to detect changes in the molecular structure of collagen glycosylation *in vivo*	1. The experimental method (acellular dermal matrix) is not applicable on human skin	(94)
Facial glycation imaging system (FGIS) to access the skin glycation index (SGI)	Human facial skin	1. Corrects for the errors caused by skin complexion 2. The first measurement system for human facial glycosylation 3. Convenient, real-time, fast, *in situ* tracking monitoring	1. Non-fluorescent AGEs cannot be detected 2. It may not be accurate enough to remove the pigmented areas	(88)
Enzyme-linked immunosorbent assay (ELISA)	Serum, urine, tissue	1. Simple, fast, cheap, and no complicated laboratory equipment required	1. The accuracy of the results depends on the specificity of the kit card antibody and the technical staff’s proficiency 2. Only one target can be tested at a time	(72)

The autofluorescence reader (AFR), a real-time, non-invasive instrument for measuring the skin AGE content by using skin autofluorescence spectroscopy, was developed by Meerwaldt et al. (91). According to the determination of the content of fluorescent AGEs in skin biopsies by AFR and HPLC, the data are obviously related. However, the AFR instrument cannot detect non-fluorescent AGEs, cannot rule out the interference of other fluorophores in the skin, and the accuracy of the results is affected by the skin colour depth.

A multiphoton microscope (MPAF) was used by Ghazaryan et al. (92) to scan skin tissue in depth and laterally to extract multiphoton autofluorescence, second harmonic generation (SHG) intensity and spectral data information. Combining two-photon microscopy and spectroscopic analysis technology, MPAF can non-invasively locate, image and quantify skin glycosylated tissue, providing real-time monitoring of the spatial and temporal effects of glycation on skin tissue.

Confocal Raman spectroscopy (CRS) was used by Pereira et al. (93) to determine the presence and differences in AGE content by identifying the reference spectra of the main AGEs present in human skin. This method can measure the content of AGEs in full-thickness skin tissue and explore the molecular mechanism of skin glycation through temporal and spatial spectral changes.

Raman signal spectroscopy was used by Shi et al. (94) to study the effect of collagen glycation. This method examines collagen fibres after decellularisation of the dermal matrix in mice and detects changes in collagen caused by non-enzymatic glycation. It is also the first research method to detect changes in the molecular structure of collagen after glycation *in vivo*.

The facial glycation imaging system (FGIS) was developed by Lee et al. (88) for the determination of the human facial skin glycation index. After the system collects facial images, it uses image analysis algorithms to calculate the autofluorescence of AGEs and the total skin reflectance to give the skin glycation index. It is also the first measurement method that is not limited to the forearm, allowing for the measurement of AGEs on human facial skin.

The above methods are primarily established on the basis of spectroscopy, whereas ELISA is widely used to study biological samples (the activity and spectra of tissue, etc.) (72). This method is relatively simple and inexpensive and does not require complicated experimental equipment. However, this method is invasive, and its accuracy depends on the experimenter and the specificity of the antibodies used in commercial kits. In addition, only one target can be tested at a time.

## Pathways Inhibiting Advanced Glycation End Products

Globally, researchers are developing AGE inhibitors using different methods to improve various chronic metabolic diseases and skin complications caused by AGEs (95, 96). The inhibition of AGEs is divided into three strategies. The first reduces AGE production by inhibiting the AGE formation pathway and prevents the damage of AGEs to the skin at the source. The second reduces the accumulation of AGEs in human tissues by catabolizing and removing the generated AGEs (61); the third disrupts the signalling mediated by the AGE-RAGE axis.

### Inhibitory Pathway Before Advanced Glycation End Products Formation

**FIGURE 5:**
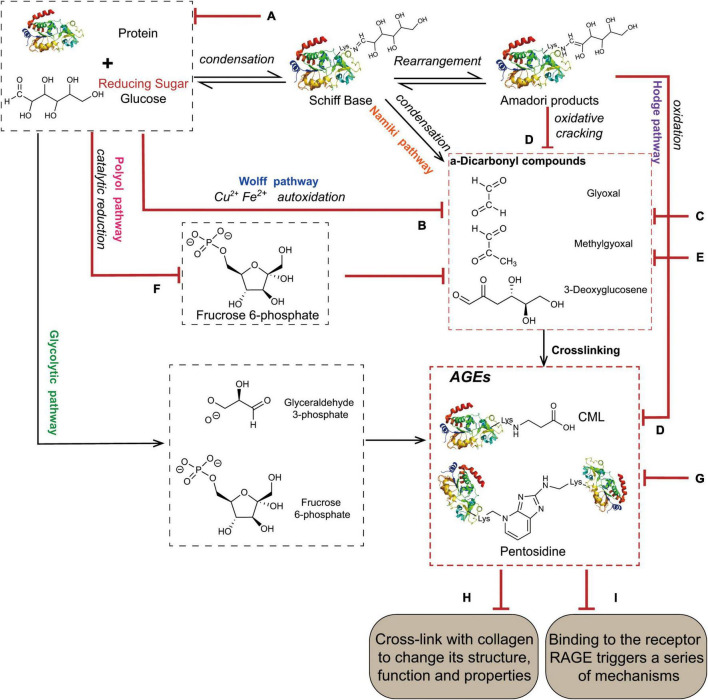
The mechanisms of AGEs inhibition. **(A)** Maintain and stabilize the protein structure. **(B)** Chelation of transition metals. **(C)** Capture and block dicarbonyl compounds. **(D)** Neutralize, inhibit, and scavenge oxidative free radicals. **(E)** Activation of the glyoxalase detoxification system. **(F)** Inhibition of aldose reductase. **(G)** Activation of the proteolytic system. **(H)** Regulation of AGE-RAGE signal transduction. **(I)** Disruption of protein cross-linking.

At present, the inhibition mode and potential action sites of the AGE formation pathway mainly include seven methods (as shown in Figure 5 and Table 3 ABCDEFG): A. maintain and stabilise the protein structure; B. chelation of transition metals; C. capture and block dicarbonyl compounds; D. neutralise, inhibit and scavenge free radicals; E. activation of the glyoxalase detoxification system; and F. inhibition of aldose reductase.

**TABLE 3:** Compounds that inhibit the formation of advanced glycation end products (AGEs) and their mechanisms based on the categories of inhibitory pathways.

Category	Inhibitory pathway	Substance	Inhibitory mechanism	References
A	Maintain and stabilise the protein structure	Phytosterols	Interacts with lysine residues of BSA	(100)
		Anthraquinones	Interacts with amino acid residues in HSA to maintain protein structure	(101)
		Folic acid	Binds to HSA and is stabilised by hydrophobic interactions and hydrogen bonding	(102)
B	Chelation of transition metals	Pyridoxamine	Binds metal ions to form complexes and inhibits oxidative degradation steps after the generation of Amadori products	(105)
		PTB, PMTB	Chelated with Cu^2+^ ions	(106)
C	Capture and block dicarbonyl compounds	Aminoguanidine	Nucleophilic addition reaction captures carbonyl groups formed by oxidative cleavage of Amadori products and prevents rearrangement and degradation	(109)
		Phloridzin	Capture MGO and GO via groups at the 3 and 5 positions of ring A	(112)
		ECG	The hydroxyl group on the A ring traps MGO to form an ECG-MGO adduct	(111)
		Quercetin	The C-6 and C-8 positions of the A ring trap MGO to scavenge dicarbonyl compounds	(113)
		Carnosine	Reduces the contents of CML and pentoglycoside in skin by reducing the number of MGO reactive groups	(110)
D	Neutralise, inhibit and scavenge free radicals	Lotus seedpod	Antioxidant properties	(116, 117)
		Milk thistle	Powerful antioxidant properties, reducing ROS formation	(119)
		Red maple leaf phenolic extract	Reduces MGO-induced oxidative stress in HaCaT cells	(120)
E	Activation of the glyoxalase detoxification system	Pterostilbene	Increases the expression level of GLO-1 and increases the content of GSH to activate the glyoxalase defence system	(121)
		*L. erythrorhizon* root	Upregulation of GLO-1 and GSH synthesis genes activates the glyoxalase system	(122)
F	Inhibition of AR	Pumpkin polysaccharide	Hydrogen bonds interact with residues on the enzyme side chains, and ionic bonds interact with the positively charged nicotinamide ring on the coenzyme	(125)
		Naringenin	It binds to the NADPH binding site of AR to form a stable complex, and interacts with the key residues Trp20 and His 10 of AR to inhibit AR activity	(126)
G	Activation of the proteolytic system	*N. alba* flower extract	Removal and recycling of accumulated AGEs in the skin as an autophagy agonist	(87)
H	Regulation of AGE-RAGE signalling	DNA aptamers	Inhibits the binding of AGEs to RAGE and blocks AGE-RAGE signalling	(131)
		Resveratrol	Significantly reduces RAGE expression by activating PPAR-γ and upregulates SR-A	(132)
		*B. ceiba L.* calyx	Regulation of RAGE expression reduces oxidative stress	(133)
		Curcumin	Inhibits ERK activity and upregulates PPAR-γ to induce AGE-R1 expression	(136)
I	Disrupt protein cross-links	Chebulic acid (CA)	Inhibit the cross-linking of AGEs with collagen and disrupts the collagen cross-linked structure	(137)
		Seaweed extract	Fragmentation of AGEs and collagen cross-links	(138)

#### A. Maintain and Stabilise the Protein Structure

The free Lys or Arg residues of proteins and glucose carbonyl carbonylamines condense to form Schiff bases, which are subsequently rearranged by an Amadori reaction to form more stable products and finally form AGEs (97). Among them, lysine residues are the sites most prone to glycation in proteins, and sugars have higher affinity for arginine, cysteine and lysine residues (98, 99). Some natural compounds inhibit AGE production by stabilising the protein structure through competitive binding to the glycation site of the protein. Phytosterols (PS) inhibit AGE formation by interacting with the lysine residues of bovine serum albumin (BSA), preventing the binding of reducing sugars to proteins (100). Liu et al. (101) showed that anthraquinones interact with amino acid residues in human serum albumin (HAS), maintain the protein structure, and inhibit the glycation reaction induced by MGO and GO. Folic acid (FA) binds to HSA, is stabilised by hydrophobic interactions and hydrogen bonds, and exhibits significant anti-glycation activity (102).

#### B. Chelation of Transition Metals

The metal ions Fe^2+^, Cu^2+^, and Fe^3+^ with catalytic oxidation activities catalyse the oxidation of proteins and other substances and generate free radicals through a process mediated by glucose, which significantly increases the rate of AGE accumulation (103). For example, the auto-oxidation of glucose catalysed by metal ions generates α-dicarbonyl compounds via the Wolff pathway (104). Pyridoxamine (PM) inhibits the oxidative degradation step after Amadori products are formed by binding metal ions to generate complexes, reducing AGE production (105). Price et al. (106) showed that common AGE inhibitors used *in vitro*, such as phenacylthiazolium bromide (PTB) and phenacyldimethylthiazolium bromide (PMTB), display significant Cu^2+^ chelation activity, which is the main mechanism for inhibiting the formation of AGEs.

#### C. Capture and Block Dicarbonyl Compounds

Glycosylated carbonyl intermediates are a class of compounds responsible for the formation of most AGEs and ALEs, and are important intermediates of Wolff pathway, polyol pathway and Namiki pathway, as well as the precursor structure of AGEs such as CML and CEL, which determines the amount of endogenous AGEs that forms (3). The reactivity is much higher than that of glucose and is associated with a high degree of physiological damage called carbonyl stress, which is collectively referred to as RCS (107, 108). Aminoguanidine (AG) (109) captures the carbonyl group formed by oxidative cleavage of Amadori products through nucleophilic addition reaction and prevents rearrangement and subsequent degradation. Carnosine reduces the contents of CML and pentoglycoside in skin by reducing the number of MGO reactive groups (110). Many studies have shown that the anti-glycation activity of flavonoids is strongly correlated with its molecular structure. The number and position of hydroxyl groups on the A and B rings, namely, 3′-, 4′ -,5′- and 7 hydroxyl groups, improve the anti-glycation activity. Epicatechin gallate (ECG) captures MGO through the hydroxyl groups on ring A to form an ECG-MGO adduct that inhibits glycation (111). The three and five positions of the phloside A ring (112) and the C-6 and C-8 positions of the quercetin A ring (113) are the main active centres for scavenging the dicarbonyl compounds MGO and GO.

#### D. Neutralise, Inhibit, and Scavenge Oxidative Free Radicals

Oxidative radicals are the most important participants in glycation reactions. Antioxidants protect the protein structure from damage and inhibit the highly reactive precursor compounds of AGEs (carbonyl compounds) that result from sugar chain cracking or lipid peroxidation. Neutralising, inhibiting, and scavenging free radicals, and reducing oxidative stress and ROS production are important approaches to inhibit AGEs (114, 115). According to published data, polyphenols are common anti-glycation active substances, and their ability to inhibit glycation reactions is related to their antioxidant properties (116). Wu et al. showed that the antioxidant activity of lotus seedpod and its metabolites was positively correlated with its antioxidant capacity, and antioxidant activity may be the basis of the AGE inhibitory effect (116, 117). Studies have shown that red grape skin extract (RGSE) (118) may prevent oxidative damage to proteins and reduce the formation of AGEs through its powerful antioxidant properties, reducing ROS formation. Milk thistle flower may reduce the CML content of skin explants by scavenging free radicals and reduce skin wrinkles (119). The red maple leaf phenolic extract possesses anti-glycation activity, reduces MGO-induced oxidative stress in HaCaT cells, and protects the skin (120).

#### E. Activation of the Glyoxalase Detoxification System

The glyoxalase system is a metabolic pathway that exists in epidermal keratinocytes and dermal fibroblasts and consists of glyoxalase I, glyoxalase II, and reduced glutathione as a cofactor. Glyoxalase I catalyses the transformation of dicarbonyl compounds such as MGO, GO, 3-DG, and glutathione into D-lactyl glutathione, which is converted into the non-toxic molecule D-lactate that is excreted from the body under the action of glyoxalase II. Glyoxalase detoxification system inhibit carbonyl stress and maintain the low tolerance level of dicarbonyl compounds. It is a powerful defence system against glycation reactions in the human body (63, 64). Studies have shown that *Pterostylus membranaceus* (PTS) (121) and *Lithospermum erythrorhizon* root (122) activate the glyoxalase defence system by upregulating the glyoxalase I (GLO-1) expression level and increasing the glutathione (GSH) content, thus reducing the production of AGEs and improving the skin condition.

#### F. Inhibition of Aldose Reductase

Aldose reductase (AR) is a key and rate-limiting enzyme in the polyol pathway. In the polyol pathway, glucose is catalytically reduced to sorbitol, converted to fructose and its metabolites, which are more reactive in glycation, and subsequently rapidly converted to α-dicarbonyl compounds (123). Therefore, controlling the flux of the polyol pathway by inhibiting AR is one of the effective methods to reduce the formation of AGEs. The isolation of chemicals with AR inhibitory activity from plant extracts is the focus of many researchers (124). Pumpkin polysaccharide contains a functional carboxyl group necessary to inhibit AR, interacts with residues on the side chain of the enzyme through its own hydrogen bonds, and interacts with the positively charged nicotinamide ring on the coenzyme through ionic bonds to finally inhibit AR and reduce the formation of AGEs (125). Naringenin binds to the NADPH binding site of AR to form a stable complex, and interacts with the key residues Trp20 and His 10 of AR to inhibit the reactivity of AR (126).

### Inhibitory Pathway After the Formation of Advanced Glycation End Products

For exogenous AGEs ingested by people through the diet, air and other routes, as well as endogenous AEGs formed by various pathways in the body, the accumulation of AGEs in human tissues is usually reduced by catabolism and elimination (61), or the signal transmission and protein cross-linking mediated by the AGE-RAGE axis are destroyed (127). These processes interrupt and inhibit AGE formation and subsequent damage to the skin. The inhibitory mode and mechanism include the following three methods: G. activation of the proteolytic system; H. regulation of AGE-RAGE signal transduction; and I. disruption of protein cross-linking.

#### G. Activation of the Proteolytic System

The UPS and autophagy are known to effectively remove and degrade damaged and misfolded proteins (67, 68). The UPS plays an important role in protein quality control and is responsible for maintaining normal cellular functions by removing damaged proteins. AGEs are often involved in the formation of cross-linked and aggregated proteins and directly inhibit UPS activity (67). Laughlin et al. (87) showed that an Nymphaea *alba* flower extract, which is an autophagy agonist, removed and recovered AGEs that accumulated in the skin, thereby improving dull and ageing skin.

#### H. Regulation of AGE-RAGE Signal Transduction

The first pathway by which AGEs damage skin is by binding to RAGE, activating P13K-AKT, MAPK-ERK, JAK2-STAT1 and other signalling pathways, and inducing the expression of NK-κB, FOXO1 and a large number of pro-inflammatory, and pro-apoptotic factors, such as TNF-α, IL-6, and IL-1β. The expression of RAGE in keratinocytes is closely related to persistent acute skin inflammation (128) and induces skin cell apoptosis, skin injury, and senescence (129). Therefore, the identification of RAGE as a therapeutic target to reduce the harm of AGEs to skin is of great importance and has broad research prospects.

Aptamers are short single-stranded DNA or RNA molecules that bind with high affinity and specificity to a variety of target proteins (130). Yamagishi et al. (131) showed that specific DNA aptamers of AGEs dose-dependently inhibit AGE-RAGE binding, thus blocking the AGE-RAGE signal. However, its safety and efficacy as a therapeutic tool require further exploration.

Regulation of RAGE expression by phytochemicals is a common method. Resveratrol significantly decreases RAGE expression and upregulates AGE scavenger receptor A (SR-A) expression by activating PPAR-γ, thus blocking AGE-RAGE signalling (132). *Bombax ceiba* L. calyx regulates the expression of RAGE and reduces the oxidative stress response, thus ameliorating the cellular dysfunction caused by AGEs (133).

Soluble RAGE (SRAGE), including AGE-R1/OST-48, AGE-R3/Galectin-3 and some scavenger receptors (MSR-AII, MSR-Bi, and CD36), have also been detected in the circulation and body fluids. AGE-R1 is considered the receptor mediating AGE turnover and clearance (134). These receptors compete with RAGE to block the range of effects of AGE-RAGE signalling on the skin (135). Lin et al. (136) found that curcumin induces AGE-R1 expression by inhibiting ERK activity and upregulating PPAR-γ, and it reduces some of the harmful effects induced by AGEs.

#### I. Disruption of Protein Cross-Linking

Advanced glycation end products form and bind to long-lived proteins in the skin, cross-linking them, damaging their structure, deforming their fibres, and eventually resulting in a loss of their biological properties. Therefore, further skin damage caused by AGEs may be reduced by investigating AGE-collagen cross-linking inhibitors and rupture agents. Studies have shown that chebulic acid (CA) (137) both inhibits age-collagen cross-linking and disrupts the cross-linked structure, but the mechanism of action remains unclear. Phenolic and tannic compounds in marine algae significantly inhibit the formation of AGEs and break the cross-links between AGEs and collagen (138).

### Different Pathways Can Inhibit Advanced Glycation End Product Accumulation

Advanced glycation end product inhibitors are divided into two main categories: synthetic and natural inhibitors. The chemical synthesised inhibitors include aminoguanidine, quinine, TZDS, and metformin, which inhibit AGEs; however, these compounds are associated many side effects and safety issues, such as reduced liver function, anaemia, vomiting, gastrointestinal disease, diarrhoea, dizziness, headache, flu and lupus symptoms, and anti-neutrophil cytoplasmic antibody-associated vasculitis (139). The inhibition of AGEs by natural bioactive substances has aroused the interest of researchers worldwide. These substances are harmless, have fewer side effects and less toxicity, and enable the use of technological progress, making them the research direction with the most potential and trend of AGE inhibitors (140).

According to published data on AGE inhibitors, most of the inhibitory mechanisms are attributed to chelation by transition metals, blocking the capture of highly active dicarbonyl compounds, and neutralising the suppression of oxygen free radicals. Few reports have assessed methods to treat AGEs that have been formed. In the early literature, AGEs were presumed to be unable to be removed and irreversible after they were generated. However, in recent years, preliminary progress has been achieved in developing strategies to eliminate the harm caused by AGEs. Research in this direction is also currently an urgent need and a promising research direction.

At present, in the exploration and development of AGE inhibitors, some researchers have confirmed that the inhibitor exerts a positive effect on related skin diseases or conditions by inhibiting the glycation reaction, such as ameliorating systemic lupus erythematosus, skin complications of diabetes, and systemic scleroderma, delaying ageing, and accelerating wound healing. AGE inhibitors are expected to be a new therapeutic agent to improve the skin condition of people and the skin complications of chronic metabolic diseases.

## Conclusion

In recent years, AGEs have been studied mainly as markers of diseases such as diabetes and key substances in the theory of carbonyl stress ageing. Comprehensive studies have shown that AGEs not only reduce skin elasticity, accumulate pigments, and produce appearance changes such as wrinkles but also destroy the skin barrier, cause the apoptosis of skin-related cells, and induce inflammation. In addition, AGEs are irreversible and difficult to metabolise. As AGEs accumulate year-by-year, the skin undergoes profound changes from the inside to the outside.

By examining the molecular mechanisms of ageing in different skin structures, we found that the skin is affected mainly through two aspects. The first is through cross-linking with long-lived proteins, which destroys the protein structure, deforms fibres, and ultimately causes the protein to lose its biological function. The second is via a series of signalling pathways mediated by AGEs binding to RAGEs, which then regulates gene expression.

There have been many studies on AGEs, but most of them have been in the context of diabetes, atherosclerosis, and liver diseases. People have gradually discovered that the impact of AGEs on the skin should not be underestimated, but research on the mechanisms of these effects is relatively scattered. This article summarises the formation and characteristics of AGEs, focussing on the molecular mechanisms by which AGEs affect the composition and structure of normal skin substances at different skin layers and induce skin issues. Based on the many skin problems induced by AGEs and the complicated mechanism, we also identified prevention and inhibition pathways, discussed systematic and comprehensive methods for measuring the content of AGEs in human skin, and summarised and analysed their advantages and disadvantages. This paper helps researchers acquire a deeper understanding of the relationship between AGEs and the skin and provides a basis for the development of effective ingredients that inhibit glycation.

## Author Contributions

FY and HM: conceptualisation. CC, LL, and J-QZ: writing–original draft preparation. MG, YH and YD: editing. All authors have read and agreed to the published version of the manuscript.

## Conflict of Interest

The authors declare that the research was conducted in the absence of any commercial or financial relationships that could be construed as a potential conflict of interest.

## Publisher’s Note

All claims expressed in this article are solely those of the authors and do not necessarily represent those of their affiliated organizations, or those of the publisher, the editors and the reviewers. Any product that may be evaluated in this article, or claim that may be made by its manufacturer, is not guaranteed or endorsed by the publisher.

## References

[1] Maillard LC. Action des acides amines sur les sucres; formation des melanoidines par voie methodique. *Comptes R Acad Sci (Paris).* (1912) 154:66–8.

[2] Ahmad S Khan H Siddiqui Z Khan MY Rehman S Shahab U AGES, Rages and s-Rage; friend or foe for cancer. *Semin Cancer Biol.* (2018) 49:44–55. 10.1016/j.semcancer.2017.07.001 28712719

[3] Singh R Barden A Mori T Beilin L. Advanced glycation end-products: a review. *Diabetologia.* (2001) 44:129–46. 11270668 10.1007/s001250051591

[4] Rasool M Malik A Butt TT Ashraf MAB Rasool R Zahid A Implications of advanced oxidation protein products (AOPPs), advanced glycation end products (AGEs) and other biomarkers in the development of cardiovascular diseases. *Saudi J Biol Sci.* (2019) 26:334–9. 10.1016/j.sjbs.2018.08.024 31485173 PMC6717110

[5] Gul A Rahman MA Salim A Simjee SU. Advanced glycation end products in senile diabetic and nondiabetic patients with cataract. *J Diabetes Complications.* (2009) 23:343–8. 10.1016/j.jdiacomp.2008.04.001 18508288

[6] Li J Liu D Sun L Lu Y Zhang Z. Advanced glycation end products and neurodegenerative diseases: mechanisms and perspective. *J Neurol Sci.* (2012) 317:1–5. 10.1016/j.jns.2012.02.018 22410257

[7] Rungratanawanich W Qu Y Wang X Essa MM Song B-J. Advanced glycation end products (AGEs) and other adducts in aging-related diseases and alcohol-mediated tissue injury. *Exp Mol Med.* (2021) 53:168–88. 10.1038/s12276-021-00561-7 33568752 PMC8080618

[8] Monnier VM Cerami A. Nonenzymatic browning *in vivo*: possible process for aging of long-lived proteins. *Science.* (1981) 211:491–3. 6779377 10.1126/science.6779377

[9] de Macedo GM Nunes S Barreto T. Skin disorders in diabetes mellitus: an epidemiology and physiopathology review. *Diabetol Metab Syndr.* (2016) 8:63. 10.1186/s13098-016-0176-y 27583022 PMC5006568

[10] Qing C. The molecular biology in wound healing & non-healing wound. *Chin. J. Traumatol.* (2017) 20:189–93. 10.1016/j.cjtee.2017.06.001 28712679 PMC5555286

[11] Ohshima H Oyobikawa M Tada A Maeda T Takiwaki H Itoh M Melanin and facial skin fluorescence as markers of yellowish discoloration with aging. *Skin Res Technol.* (2009) 15:496–502. 10.1111/j.1600-0846.2009.00396.x 19832964

[12] Atzeni IM Boersema J Pas HH Diercks GFH Scheijen JLJM Schalkwijk CG Is skin autofluorescence (SAF) representative of dermal advanced glycation endproducts (AGEs) in dark skin? A pilot study. *Heliyon.* (2020) 6:e05364. 10.1016/j.heliyon.2020.e05364 33241137 PMC7674296

[13] Yoshinaga E Kawada A Ono K Fujimoto E Wachi H Harumiya S N(ε)-(carboxymethyl)lysine modification of elastin alters its biological properties: implications for the accumulation of abnormal elastic fibers in actinic elastosis. *J. Investig. Dermatol.* (2012) 132:315–23. 10.1038/jid.2011.298 21956123

[14] Emel’yanov VV. [Glycation, antiglycation and deglycation: role in mechanisms of aging ang geroprotective (literature review)]. *Adv Gerontol.* (2016) 29:407–16. 28525687

[15] Horiuchi S Araki N Morino Y. Immunochemical approach to characterize advanced glycation end products of the maillard reaction. evidence for the presence of a common structure. *J Biol Chem.* (1991) 266:7329–32. 2019568

[16] Wang X-J Ma S-B Liu Z-F Li H Gao W-Y. Elevated levels of α-dicarbonyl compounds in the plasma of type ii diabetics and their relevance with diabetic nephropathy. *J Chromatogr B Analyt Technol Biomed Life Sci.* (2019) 1106–7:19–25. 10.1016/j.jchromb.2018.12.027 30639946

[17] Wolff SP Bascal ZA Hunt JV. “Autoxidative glycosylation”: free radicals and glycation theory. *Prog Clin Biol Res.* (1989) 304:259–75. 2675032

[18] Namiki M. [Advances in the maillard reaction and glycation researches–mainly on the Namiki pathway]. *Seikagaku.* (2003) 75:37–42. 12645131

[19] Namiki M Hayashi T editors. A new mechanism of the maillard reaction involving sugar fragmentation and free radical formation. In: *Proceedings of the ACS Symposium Series.* Washington, DC: American Chemical Society (1983).

[20] Hamada Y Araki N Horiuchi S Hotta N. Role of polyol pathway in nonenzymatic glycation. *Nephrol Dial Transplant.* (1996) 11(Suppl. 5):95–8. 10.1093/ndt/11.supp5.95 9044317

[21] Luevano-Contreras C Garay-Sevilla ME Chapman-Novakofski K. Role of dietary advanced glycation end products in diabetes mellitus. *J Evid Based Complement Altern Med.* (2013) 18:50–66.

[22] Ott C Jacobs K Haucke E Navarrete Santos A Grune T Simm A. Role of advanced glycation end products in cellular signaling. *Redox Biol.* (2014) 2:411–29. 10.1016/j.redox.2013.12.016 24624331 PMC3949097

[23] Sirangelo I Iannuzzi C. Understanding the role of protein glycation in the amyloid aggregation process. *Int J Mol Sci.* (2021) 22:6609. 10.3390/ijms22126609 PMC8235188 34205510

[24] Monnier VM Sell DR. Prevention and repair of protein damage by the maillard reaction *in vivo*. *Rejuvenation Res.* (2006) 9:264–73. 16706654 10.1089/rej.2006.9.264

[25] Chuyen NV. Toxicity of the ages generated from the Maillard reaction: on the relationship of food-ages and biological-ages. *Mol Nutr Food Res.* (2006) 50:1140–9. 10.1002/mnfr.200600144 17131455

[26] Gill V Kumar V Singh K Kumar A Kim JJ. Advanced glycation end products (AGEs) may be a striking link between modern diet and health. *Biomolecules.* (2019) 9:888. 10.3390/biom9120888 31861217 PMC6995512

[27] Perrone A Giovino A Benny J Martinelli F. Advanced glycation end products (AGEs): biochemistry, signaling, analytical methods, and epigenetic effects. *Oxid Med Cell Longev.* (2020) 2020:3818196. 10.1155/2020/3818196 32256950 PMC7104326

[28] Kellow NJ Coughlan MT Reid CM. Association between habitual dietary and lifestyle behaviours and skin autofluorescence (SAF), a marker of tissue accumulation of advanced glycation endproducts (AGEs), in healthy adults. *Eur J Nutr.* (2018) 57:2209–16. 10.1007/s00394-017-1495-y 28656390

[29] Xin C Wang Y Liu M Zhang B Yang S. Correlation analysis between advanced glycation end products detected noninvasively and skin aging factors. *J Cosmet Dermatol.* (2021) 20:243–8. 10.1111/jocd.13452 32333482

[30] Verzijl N DeGroot J Thorpe SR Bank RA Shaw JN Lyons TJ Effect of collagen turnover on the accumulation of advanced glycation end products. *J Biol Chem.* (2000) 275:39027–31. 10.1074/jbc.M006700200 10976109

[31] Jud P Sourij H. Therapeutic options to reduce advanced glycation end products in patients with diabetes mellitus: a review. *Diabetes Res Clin Pract.* (2019) 148:54–63. 10.1016/j.diabres.2018.11.016 30500546

[32] Simó-Servat O Planas A Ciudin A Simó R Hernández C. Assessment of advanced glycation end-products as a biomarker of diabetic outcomes. *Endocrinol Diabetes Nutr (Engl Ed).* (2018) 65:540–5. 10.1016/j.endinu.2018.06.003 30077632

[33] Oleniuc M Schiller A Secara I Onofriescu M Hogas S Apetrii M Evaluation of advanced glycation end products accumulation, using skin autofluorescence, in CKD and dialysis patients. *Int Urol Nephrol.* (2012) 44:1441–9. 10.1007/s11255-011-0097-5 22160646

[34] Chilelli NC Burlina S Lapolla A. Ages, rather than hyperglycemia, are responsible for microvascular complications in diabetes: a “glycoxidation-centric” point of view. *Nutr Metab Cardiovasc Dis.* (2013) 23:913–9. 10.1016/j.numecd.2013.04.004 23786818

[35] Vlassara H Uribarri J. Advanced glycation end products (AGE) and diabetes: cause, effect, or both? *Curr Diab Rep.* (2014) 14:453. 10.1007/s11892-013-0453-1 24292971 PMC3903318

[36] Ruiz HH Ramasamy R Schmidt AM. Advanced glycation end products: building on the concept of the “common soil” in metabolic disease. *Endocrinology.* (2020) 161:bqz006. 10.1210/endocr/bqz006 31638645 PMC7188081

[37] Oleniuc M Secara I Onofriescu M Hogas S Voroneanu L Siriopol D Consequences of advanced glycation end products accumulation in chronic kidney disease and clinical usefulness of their assessment using a non-invasive technique – skin autofluorescence. *Maedica (Bucur).* (2011) 6:298–307. 22879845 PMC3391948

[38] Meerwaldt R Hartog JWL Graaff R Huisman RJ Links TP den Hollander NC Skin autofluorescence, a measure of cumulative metabolic stress and advanced glycation end products, predicts mortality in hemodialysis patients. *J Am Soc Nephrol.* (2005) 16:3687–93. 16280473 10.1681/ASN.2005020144

[39] Arsov S Graaff R van Oeveren W Stegmayr B Sikole A Rakhorst G Advanced glycation end-products and skin autofluorescence in end-stage renal disease: a review. *Clin Chem Lab Med.* (2014) 52:11–20. 10.1515/cclm-2012-0832 23612551

[40] Salazar J Navarro C Ortega Á Nava M Morillo D Torres W Advanced glycation end products: new clinical and molecular perspectives. *Int J Environ Res Public Health.* (2021) 18:7236. 10.3390/ijerph18147236 34299683 PMC8306599

[41] França RDA Esteves ADBA Borges CDM Quadros KRDS Falcão LCN Caramori JCT Advanced glycation end-products (AGEs) accumulation in skin: relations with chronic kidney disease-mineral and bone disorder. *J Bras Nefrol.* (2017) 39:253–60. 10.5935/0101-2800.20170042 28902232

[42] Papagrigoraki A Del Giglio M Cosma C Maurelli M Girolomoni G Lapolla A. Advanced glycation end products are increased in the skin and blood of patients with severe psoriasis. *Acta Derm Venereol.* (2017) 97:782–7. 10.2340/00015555-2661 28358411

[43] Uruska A Gandecka A Araszkiewicz A Zozulinska-Ziolkiewicz D. Accumulation of advanced glycation end products in the skin is accelerated in relation to insulin resistance in people with type 1 diabetes mellitus. *Diabet Med.* (2019) 36:620–5. 10.1111/dme.13921 30706538

[44] Yu Y Thorpe SR Jenkins AJ Shaw JN Sochaski MA McGee D Advanced glycation end-products and methionine sulphoxide in skin collagen of patients with type 1 diabetes. *Diabetologia.* (2006) 49:2488–98. 10.1007/s00125-006-0355-8 16955213

[45] Nienhuis HL de Leeuw K Bijzet J Smit A Schalkwijk CG Graaff R Skin autofluorescence is increased in systemic lupus erythematosus but is not reflected by elevated plasma levels of advanced glycation endproducts. *Rheumatology (Oxford).* (2008) 47:1554–8. 10.1093/rheumatology/ken302 18701539

[46] Nowak A Przywara-Chowaniec B Damasiewicz-Bodzek A Blachut D Nowalany-Kozielska E Tyrpien-Golder K. Advanced glycation end-products (AGEs) and their soluble receptor (sRAGE) in women suffering from systemic lupus erythematosus (SLE). *Cells.* (2021) 10:3523. 10.3390/cells10123523 34944030 PMC8700453

[47] Atzeni IM van de Zande SC Westra J Zwerver J Smit AJ Mulder DJ. The age reader: a non-invasive method to assess long-term tissue damage. *Methods.* (2021) S1046–2023(21)00059-1. 10.1016/j.ymeth.2021.02.016 33636313

[48] Moldogazieva NT Mokhosoev IM Mel’nikova TI Porozov YB Terentiev AA. Oxidative stress and advanced lipoxidation and glycation end products (ALEs and AGEs) in aging and age-related diseases. *Oxid Med Cell Longev.* (2019) 2019:3085756. 10.1155/2019/3085756 31485289 PMC6710759

[49] Hudson BI Lippman ME. Targeting rage signaling in inflammatory disease. *Annu Rev Med.* (2018) 69:349–64. 10.1146/annurev-med-041316-085215 29106804

[50] Bilova T Paudel G Shilyaev N Schmidt R Brauch D Tarakhovskaya E Global proteomic analysis of advanced glycation end products in the arabidopsis proteome provides evidence for age-related glycation hot spots. *J Biol Chem.* (2017) 292:15758–76. 10.1074/jbc.M117.794537 28611063 PMC5612108

[51] Sergi D Boulestin H Campbell FM Williams LM. The role of dietary advanced glycation end products in metabolic dysfunction. *Mol Nutr Food Res.* (2021) 65:e1900934. 10.1002/mnfr.201900934 32246887

[52] Chen J Waqas K Tan RC Voortman T Ikram MA Nijsten TEC The association between dietary and skin advanced glycation end products: the rotterdam study. *Am J Clin Nutr.* (2020) 112:129–37. 10.1093/ajcn/nqaa117 32453418 PMC7326595

[53] Zhang Q Wang Y Fu L. Dietary advanced glycation end-products: perspectives linking food processing with health implications. *Compr Rev Food Sci Food Saf.* (2020) 19:2559–87. 10.1111/1541-4337.12593 33336972

[54] Cerami C Founds H Nicholl I Mitsuhashi T Giordano D Vanpatten S Tobacco smoke is a source of toxic reactive glycation products. *Proc Natl Acad Sci USA.* (1997) 94:13915–20. 9391127 10.1073/pnas.94.25.13915 PMC28407

[55] Dickerson TJ Janda KD. A previously undescribed chemical link between smoking and metabolic disease. *Proc Natl Acad Sci USA.* (2002) 99:15084–8. 12403823 10.1073/pnas.222561699 PMC137547

[56] Kellow NJ Coughlan MT Reid CM. Association between habitual dietary and lifestyle behaviours and skin autofluorescence (SAF), a marker of tissue accumulation of advanced glycation endproducts (AGEs), in healthy adults. *Eur J Nutr.* (2017) 57:2209–16. 10.1007/s00394-017-1495-y 28656390

[57] Federico G Gori M Randazzo E Vierucci F. Skin advanced glycation end-products evaluation in infants according to the type of feeding and mother’s smoking habits. *SAGE Open Med.* (2016) 4:2050312116682126. 10.1177/2050312116682126 28210490 PMC5302171

[58] Gursinsky T Ruhs S Friess U Diabate S Krug HF Silber RE Air pollution-associated fly ash particles induce fibrotic mechanisms in primary fibroblasts. *Biol Chem.* (2006) 387:1411–20. 10.1515/BC.2006.177 17081114

[59] Pageon H Poumés-Ballihaut C Zucchi H Bastien P Tancrede E Asselineau D. Aged human skin is more susceptible than young skin to accumulate advanced glycoxidation products induced by sun exposure. *J Aging Sci.* (2013) 1:112. 10.4172/2329-8847.1000112

[60] Crisan M Taulescu M Crisan D Cosgarea R Parvu A Catoi C Expression of advanced glycation end-products on sun-exposed and non-exposed cutaneous sites during the ageing process in humans. *PLoS One.* (2013) 8:e75003. 10.1371/journal.pone.0075003 24116020 PMC3792075

[61] Rowan S Bejarano E Taylor A. Mechanistic targeting of advanced glycation end-products in age-related diseases. *Biochim Biophys Acta Mol Basis Dis.* (2018) 1864:3631–43. 10.1016/j.bbadis.2018.08.036 30279139 PMC6822271

[62] Desmoulière A Bonté F Fournet M. Glycation damage: a possible hub for major pathophysiological disorders and aging. *Aging Dis.* (2018) 9:880–900. 10.14336/ad.2017.1121 30271665 PMC6147582

[63] Saeed M Kausar MA Singh R Siddiqui AJ Akhter A. The role of glyoxalase in glycation and carbonyl stress induced metabolic disorders. *Curr Protein Pept Sci.* (2020) 21:846–59. 10.2174/1389203721666200505101734 32368974

[64] Yumnam S Subedi L Kim SY. Glyoxalase system in the progression of skin aging and skin malignancies. *Int J Mol Sci.* (2020) 22:310. 10.3390/ijms22010310 33396745 PMC7794849

[65] Schalkwijk CG Stehouwer CDA. Methylglyoxal, a highly reactive dicarbonyl compound, in diabetes, its vascular complications, and other age-related diseases. *Physiol Rev.* (2020) 100:407–61. 10.1152/physrev.00001.2019 31539311

[66] Van Schaftingen E Collard F Wiame E Veiga-da-Cunha M. Enzymatic repair of amadori products. *Amino Acids.* (2012) 42:1143–50. 10.1007/s00726-010-0780-3 20967558

[67] Kastle M Grune T. Protein oxidative modification in the aging organism and the role of the ubiquitin proteasomal system. *Curr Pharm Des.* (2011) 17:4007–22. 22188451 10.2174/138161211798764898

[68] Cuervo AM. Autophagy and aging: keeping that old broom working. *Trends Genet.* (2008) 24:604–12. 10.1016/j.tig.2008.10.002 18992957 PMC2745226

[69] Korovila I Hugo M Castro JP Weber D Höhn A Grune T Proteostasis, oxidative stress and aging. *Redox Biol.* (2017) 13:550–67. 10.1016/j.redox.2017.07.008 28763764 PMC5536880

[70] Yokota M Masaki H Okano Y Tokudome Y. Effect of glycation focusing on the process of epidermal lipid synthesis in a reconstructed skin model and membrane fluidity of stratum corneum lipids. *Dermatoendocrinol.* (2017) 9:e1338992. 10.1080/19381980.2017.1338992 29484088 PMC5821160

[71] Park HY Kim JH Jung M Chung CH Hasham R Park CS A Long-standing hyperglycaemic condition impairs skin barrier by accelerating skin ageing process. *Exp Dermatol.* (2011) 20:969–74. 10.1111/j.1600-0625.2011.01364.x 22017743

[72] Lee KH Ng YP Cheah PS Lim CK Toh MS. Molecular characterization of glycation-associated skin ageing: an alternative skin model to study *in vitro* antiglycation activity of topical cosmeceutical and pharmaceutical formulations. *Br J Dermatol.* (2017) 176:159–67. 10.1111/bjd.14832 27363533

[73] Pennacchi PC de Almeida ME Gomes OL Faiao-Flores F de Araujo Crepaldi MC Dos Santos MF Glycated reconstructed human skin as a platform to study the pathogenesis of skin aging. *Tissue Eng Part A.* (2015) 21:2417–25. 10.1089/ten.TEA.2015.0009 26132636

[74] Pageon H Zucchi H Rousset F Monnier VM Asselineau D. Skin aging by glycation: lessons from the reconstructed skin model. *Clin Chem Lab Med.* (2014) 52:169–74. 10.1515/cclm-2013-0091 23770560

[75] Pageon H Asselineau D. An *in vitro* approach to the chronological aging of skin by glycation of the collagen: the biological effect of glycation on the reconstructed skin model. *Ann N Y Acad Sci.* (2005) 1043:529–32. 10.1196/annals.1333.060 16037275

[76] Lee EJ Kim JY Oh SH. Advanced glycation end products (AGEs) promote melanogenesis through receptor for ages. *Sci Rep.* (2016) 6:27848. 10.1038/srep27848 27293210 PMC4904211

[77] Zhu P Ren M Yang C Hu YX Ran JM Yan L. Involvement of RAGE, MAPK and Nf-kappaB pathways in AGEs-induced MMP-9 activation in HaCaT keratinocytes. *Exp Dermatol.* (2012) 21:123–9. 10.1111/j.1600-0625.2011.01408.x 22229442

[78] Xu X Zheng Y Huang Y Chen J Gong Z Li Y Cathepsin D contributes to the accumulation of advanced glycation end products during photoaging. *J Dermatol Sci.* (2018) 90:263–75. 10.1016/j.jdermsci.2018.02.009 29501392

[79] Alikhani M Maclellan CM Raptis M Vora S Trackman PC Graves DT. Advanced glycation end products induce apoptosis in fibroblasts through activation of ROS, MAP kinases, and the foxo1 transcription factor. *Am J Physiol Cell Physiol.* (2007) 292:C850–6. 10.1152/ajpcell.00356.2006 17005604

[80] Okano Y Masaki H Sakurai H. Dysfunction of dermal fibroblasts induced by advanced glycation end-products (AGEs) and the contribution of a nonspecific interaction with cell membrane and ages. *J Dermatol Sci.* (2002) 29:171–80. 10.1016/s0923-1811(02)00021-x 12234706

[81] Horikawa T Hiramoto K Goto K Sekijima H Ooi K. Differences in the mechanism of type 1 and type 2 diabetes-induced skin dryness by using model mice. *Int J Med Sci.* (2021) 18:474–81. 10.7150/ijms.50764 33390816 PMC7757134

[82] Asadipooya K Uy EM. Advanced glycation end products (AGEs), receptor for ages, diabetes, and bone: review of the literature. *J Endocr Soc.* (2019) 3:1799–818. 10.1210/js.2019-00160 31528827 PMC6734192

[83] Bierhaus A Humpert PM Morcos M Wendt T Chavakis T Arnold B Understanding rage, the receptor for advanced glycation end products. *J Mol Med (Berl).* (2005) 83:876–86. 16133426 10.1007/s00109-005-0688-7

[84] Bestall SM Hulse RP Blackley Z Swift M Ved N Paton K Sensory neuronal sensitisation occurs through HMGB-1-RAGE and TRPV1 in high-glucose conditions. *J Cell Sci.* (2018) 131:jcs215939. 10.1242/jcs.215939 29930087 PMC6080605

[85] Lohwasser C Neureiter D Weigle B Kirchner T Schuppan D. The receptor for advanced glycation end products is highly expressed in the skin and upregulated by advanced glycation end products and tumor necrosis factor-alpha. *J Invest Dermatol.* (2006) 126:291–9. 10.1038/sj.jid.5700070 16374460

[86] Gautieri A Passini FS Silvan U Guizar-Sicairos M Carimati G Volpi P Advanced glycation end-products: mechanics of aged collagen from molecule to tissue. *Matrix Biol.* (2017) 59:95–108. 10.1016/j.matbio.2016.09.001 27616134

[87] Laughlin T Tan Y Jarrold B Chen J Li L Fang B Autophagy activators stimulate the removal of advanced glycation end products in human keratinocytes. *J Eur Acad Dermatol Venereol.* (2020) 34(Suppl. 3):12–8. 10.1111/jdv.16453 32557807

[88] Lee J Jeong ET Lim JM Park SG. Development of the facial glycation imaging system for *in situ* human face skin glycation index measurement. *J Cosmet Dermatol.* (2021) 20:2963–8. 10.1111/jocd.13943 33522691 PMC8451778

[89] Stephen EA Venkatasubramaniam A Good TA Topoleski LD. The effect of glycation on arterial microstructure and mechanical response. *J Biomed Mater Res A.* (2014) 102:2565–72. 10.1002/jbm.a.34927 23963622

[90] Kueper T Grune T Prahl S Lenz H Welge V Biernoth T Vimentin is the specific target in skin glycation. structural prerequisites, functional consequences, and role in skin aging. *J Biol Chem.* (2007) 282:23427–36. 17567584 10.1074/jbc.M701586200

[91] Meerwaldt R Graaff R Oomen PHN Links TP Jager JJ Alderson NL Simple non-invasive assessment of advanced glycation endproduct accumulation. *Diabetologia.* (2004) 47:1324–30. 10.1007/s00125-004-1451-2 15243705

[92] Ghazaryan AA Hu PS Chen SJ Tan HY Dong CY. Spatial and temporal analysis of skin glycation by the use of multiphoton microscopy and spectroscopy. *J Dermatol Sci.* (2012) 65:189–95. 10.1016/j.jdermsci.2011.12.012 22277703

[93] Pereira AFM Rodrigues BVM Neto LPM de O Lopes L da Costa ALF Santos AS Confocal raman spectroscopy as a tool to assess advanced glycation end products on solar-exposed human skin. *Vibr Spectrosc.* (2021) 114:103234. 10.1016/j.vibspec.2021.103234

[94] Shi P Liu H Deng X Jin Y Wang Q Liu H Label-free nonenzymatic glycation monitoring of collagen scaffolds in type 2 diabetic mice by confocal raman microspectroscopy. *J Biomed Opt.* (2015) 20:27002. 10.1117/1.JBO.20.2.027002 25671672

[95] Velichkova S Foubert K Pieters L. Natural products as a source of inspiration for novel inhibitors of advanced glycation endproducts (AGEs) formation. *Planta Med.* (2021) 87:780–801. 10.1055/a-1527-7611 34341977

[96] Song Q Liu J Dong L Wang X Zhang X. Novel advances in inhibiting advanced glycation end product formation using natural compounds. *Biomed Pharmacother.* (2021) 140:111750. 10.1016/j.biopha.2021.111750 34051615

[97] Rabbani G Ahn SN. Structure, enzymatic activities, glycation and therapeutic potential of human serum albumin: a natural cargo. *Int J Biol Macromol.* (2019) 123:979–90. 10.1016/j.ijbiomac.2018.11.053 30439428

[98] Ueda Y Matsumoto H. Recent topics in chemical and clinical research on glycated albumin. *J Diabetes Sci Technol.* (2015) 9:177–82. 10.1177/1932296814567225 25614014 PMC4604594

[99] Barnaby OS Cerny RL Clarke W Hage DS. Comparison of modification sites formed on human serum albumin at various stages of glycation. *Clin Chim Acta.* (2011) 412:277–85. 10.1016/j.cca.2010.10.018 21034726 PMC3053033

[100] Sobhy R Zhan F Mekawi E Khalifa I Liang H Li B. The noncovalent conjugations of bovine serum albumin with three structurally different phytosterols exerted antiglycation effects: a study with ages-inhibition, multispectral, and docking investigations. *Bioorg Chem.* (2020) 94:103478. 10.1016/j.bioorg.2019.103478 31806157

[101] Liu W Cai A Carley R Rocchio R Seeram NP. Bioactive anthraquinones found in plant foods interact with human serum albumin and inhibit the formation of advanced glycation endproducts. *J Food Bioactives.* (2018) 4:130–8.

[102] Al Jaseem MAJ Abdullah KM Qais FA Shamsi A Naseem I. Mechanistic insight into glycation inhibition of human serum albumin by vitamin B9: multispectroscopic and molecular docking approach. *Int J Biol Macromol.* (2021) 181:426–34. 10.1016/j.ijbiomac.2021.03.153 33775768

[103] Sajithlal GB Chithra P Chandrakasan G. The role of metal-catalyzed oxidation in the formation of advanced glycation end products: an *in vitro* study on collagen. *Free Radic Biol Med.* (1998) 25:265–9. 9680171 10.1016/s0891-5849(98)00035-5

[104] Hunt JV Dean RT Wolff SP. Hydroxyl radical production and autoxidative glycosylation. glucose autoxidation as the cause of protein damage in the experimental glycation model of diabetes mellitus and ageing. *Biochem J.* (1988) 256:205–12. 2851978 10.1042/bj2560205 PMC1135388

[105] Voziyan PA Hudson BG. Pyridoxamine: the many virtues of a Maillard reaction inhibitor. *Ann N Y Acad Sci.* (2005) 1043:807–16. 10.1196/annals.1333.093 16037308

[106] Price DL Rhett PM Thorpe SR Baynes JW. Chelating activity of advanced glycation end-product inhibitors. *J Biol Chem.* (2001) 276:48967–72. 10.1074/jbc.M108196200 11677237

[107] Cho SJ Roman G Yeboah F Konishi Y. The road to advanced glycation end products: a mechanistic perspective. *Curr Med Chem.* (2007) 14:1653–71. 17584071 10.2174/092986707780830989

[108] Vistoli G De Maddis D Cipak A Zarkovic N Carini M Aldini G. Advanced glycoxidation and lipoxidation end products (AGEs and ALEs): an overview of their mechanisms of formation. *Free Radic Res.* (2013) 47(Suppl. 1):3–27. 10.3109/10715762.2013.815348 23767955

[109] Thornalley PJ. Use of aminoguanidine (pimagedine) to prevent the formation of advanced glycation endproducts. *Arch Biochem Biophys.* (2003) 419:31–40. 10.1016/j.abb.2003.08.013 14568006

[110] Narda M Peno-Mazzarino L Krutmann J Trullas C Granger C. Novel facial cream containing carnosine inhibits formation of advanced glycation end-products in human skin. *Skin Pharmacol Physiol.* (2018) 31:324–31. 10.1159/000492276 30199874 PMC6262686

[111] Wu X Zhang G Hu X Pan J Liao Y Ding H. Inhibitory effect of epicatechin gallate on protein glycation. *Food Res Int.* (2019) 122:230–40. 10.1016/j.foodres.2019.04.023 31229076

[112] Shao X Bai N He K Ho C-T Yang CS Sang S. Apple polyphenols, phloretin and phloridzin: new trapping agents of reactive dicarbonyl species. *Chem Res Toxicol.* (2008) 21:2042–50. 10.1021/tx800227v 18774823

[113] Bhuiyan MNI Mitsuhashi S Sigetomi K Ubukata M. Quercetin inhibits advanced glycation end product formation via chelating metal ions, trapping methylglyoxal, and trapping reactive oxygen species. *Biosci Biotechnol Biochem.* (2017) 81:882–90. 10.1080/09168451.2017.1282805 28388357

[114] Shin S Son D Kim M Lee S Roh K-B Ryu D Ameliorating effect of akebia quinata fruit extracts on skin aging induced by advanced glycation end products. *Nutrients.* (2015) 7:9337–52. 10.3390/nu7115478 26569300 PMC4663606

[115] Hidalgo FJ Zamora R. Interplay between the Maillard reaction and lipid peroxidation in biochemical systems. *Ann N Y Acad Sci.* (2005) 1043:319–26. 10.1196/annals.1333.039 16037254

[116] Crascì L Lauro MR Puglisi G Panico A. Natural antioxidant polyphenols on inflammation management: anti-glycation activity vs metalloproteinases inhibition. *Crit Rev Food Sci Nutr.* (2018) 58:893–904. 10.1080/10408398.2016.1229657 27646710

[117] Wu Q Li S Li X Fu X Sui Y Guo T A significant inhibitory effect on advanced glycation end product formation by catechin as the major metabolite of lotus seedpod oligomeric procyanidins. *Nutrients.* (2014) 6:3230–44. 10.3390/nu6083230 25123249 PMC4145305

[118] Jariyapamornkoon N Yibchok-Anun S Adisakwattana S. Inhibition of advanced glycation end products by red grape skin extract and its antioxidant activity. *BMC Complement Altern Med.* (2013) 13:171. 10.1186/1472-6882-13-171 23849496 PMC3716656

[119] Shin S Lee JA Kim M Kum H Jung E Park D. Anti-glycation activities of phenolic constituents from *Silybum marianum* (milk thistle) flower *in vitro* and on human explants. *Molecules.* (2015) 20:3549–64. 10.3390/molecules20033549 25706757 PMC6272457

[120] Liu C Guo H Dain JA Wan Y Gao XH Chen HD Cytoprotective effects of a proprietary red maple leaf extract and its major polyphenol, ginnalin a, against hydrogen peroxide and methylglyoxal induced oxidative stress in human keratinocytes. *Food Funct.* (2020) 11:5105–14. 10.1039/d0fo00359j 32356551 PMC10902859

[121] Tang D Xiao W Gu WT Zhang ZT Xu SH Chen ZQ Pterostilbene prevents methylglyoxal-induced cytotoxicity in endothelial cells by regulating glyoxalase, oxidative stress and apoptosis. *Food Chem Toxicol.* (2021) 153:112244. 10.1016/j.fct.2021.112244 33930484

[122] Glynn KM Anderson P Fast DJ Koedam J Rebhun JF Velliquette RA. Gromwell (*Lithospermum erythrorhizon*) root extract protects against glycation and related inflammatory and oxidative stress while offering UV absorption capability. *Exp Dermatol.* (2018) 27:1043–7. 10.1111/exd.13706 29906314

[123] Quattrini L La Motta C. Aldose reductase inhibitors: 2013-present. *Expert Opin Ther Pat.* (2019) 29:199–213. 10.1080/13543776.2019.1582646 30760060

[124] Veeresham C Rama Rao A Asres K. Aldose reductase inhibitors of plant origin. *Phytother Res.* (2014) 28:317–33. 10.1002/ptr.5000 23674239

[125] Wang X Zhang L-S Dong L-L. Inhibitory effect of polysaccharides from pumpkin on advanced glycation end-products formation and aldose reductase activity. *Food Chem.* (2012) 130:821–5. 10.1016/j.foodchem.2011.07.064

[126] Khan MS Qais FA Rehman MT Ismail MH Alokail MS Altwaijry N Mechanistic inhibition of non-enzymatic glycation and aldose reductase activity by naringenin: binding, enzyme kinetics and molecular docking analysis. *Int J Biol Macromol.* (2020) 159:87–97. 10.1016/j.ijbiomac.2020.04.226 32437808

[127] Shen CY Lu CH Wu CH Li KJ Kuo YM Hsieh SC The development of maillard reaction, and advanced glycation end product (AGE)-receptor for age (RAGE) signaling inhibitors as novel therapeutic strategies for patients with age-related diseases. *Molecules.* (2020) 25:5591. 10.3390/molecules25235591 33261212 PMC7729569

[128] Leibold JS Riehl A Hettinger J Durben M Hess J Angel P. Keratinocyte-specific deletion of the receptor rage modulates the kinetics of skin inflammation *in vivo*. *J Invest Dermatol.* (2013) 133:2400–6. 10.1038/jid.2013.185 23594597

[129] Iwamura M Yamamoto Y Kitayama Y Higuchi K Fujimura T Hase T Epidermal expression of receptor for advanced glycation end products (RAGE) is related to inflammation and apoptosis in human skin. *Exp Dermatol.* (2016) 25:235–7. 10.1111/exd.12899 26566598

[130] Guan B Zhang X. Aptamers as versatile ligands for biomedical and pharmaceutical applications. *Int J Nanomedicine.* (2020) 15:1059–71. 10.2147/IJN.S237544 32110008 PMC7035142

[131] Yamagishi S-I Taguchi K Fukami K. DNA–aptamers raised against AGEs as a blocker of various aging-related disorders. *Glycoconj J.* (2016) 33:683–90. 10.1007/s10719-016-9682-2 27338620

[132] Zhang Y Luo Z Ma L Xu Q Yang Q Si L. Resveratrol prevents the impairment of advanced glycosylation end products (AGE) on macrophage lipid homeostasis by suppressing the receptor for age via peroxisome proliferator-activated receptor gamma activation. *Int J Mol Med.* (2010) 25:729–34. 10.3892/ijmm_00000398 20372816

[133] Komati A Anand A Shaik H Mudiam MKR Suresh Babu K Tiwari AK. *Bombax ceiba* (Linn.) calyxes ameliorate methylglyoxal-induced oxidative stress via modulation of rage expression: identification of active phytometabolites by GC-MS analysis. *Food Funct.* (2020) 11:5486–97. 10.1039/c9fo02714a 32500907

[134] Zhuang A Forbes JM. Diabetic kidney disease: a role for advanced glycation end-product receptor 1 (AGE-R1)? *Glycoconj J.* (2016) 33:645–52. 10.1007/s10719-016-9693-z 27270766

[135] Merhi Z Kandaraki EA Diamanti-Kandarakis E. Implications and future perspectives of ages in pcos pathophysiology. *Trends Endocrinol Metab.* (2019) 30:150–62. 10.1016/j.tem.2019.01.005 30712978

[136] Lin J Tang Y Kang Q Chen A. Curcumin eliminates the inhibitory effect of advanced glycation end-products (AGEs) on gene expression of age receptor-1 in hepatic stellate cells *in vitro*. *Lab Invest.* (2012) 92:827–41. 10.1038/labinvest.2012.53 22449800 PMC3365656

[137] Lee J-Y Oh J-G Kim JS Lee K-W. Effects of chebulic acid on advanced glycation endproducts-induced collagen cross-links. *Biol Pharm Bull.* (2014) 37:1162–7. 24759763 10.1248/bpb.b14-00034

[138] Cho CH Youm GH Kim M Kim S Song EJ Nam YD Evaluation of the relationship between bioactive components in seaweeds and advanced glycation end-products inhibitory activities using principal component analysis. *Plant Foods Hum Nutr.* (2021) 76:326–33. 10.1007/s11130-021-00908-5 34279786

[139] Khangholi S Majid FA Berwary NJ Ahmad F Aziz RB. The mechanisms of inhibition of advanced glycation end products formation through polyphenols in hyperglycemic condition. *Planta Med.* (2016) 82:32–45. 10.1055/s-0035-1558086 26550791

[140] M V Wang K. Dietary natural products as a potential inhibitor towards advanced glycation end products and hyperglycemic complications: a phytotherapy approaches. *Biomed Pharmacother.* (2021) 144:112336. 10.1016/j.biopha.2021.112336 34678719

